# Clinical chemistry laboratory test overuse in a cardiology clinic: a single-center study

**DOI:** 10.25122/jml-2022-0338

**Published:** 2023-04

**Authors:** Abeer Alshwareb, Mostafa Rashed, Faraz Farooqi, Ibrahim Alhabib, Neethu Betty Theruvan, Omar El-Masry

**Affiliations:** 1 Department of Clinical Laboratory Sciences, College of Applied Medical Sciences, Imam Abdulrahman Bin Faisal University, Dammam, Saudi Arabia; 2 Department of Cardiac Technology, College of Applied Medical Sciences, Imam Abdulrahman Bin Faisal University, Dammam, Saudi Arabia; 3 College of Dentistry, Imam Abdulrahman Bin Faisal University, Dammam, Saudi Arabia

**Keywords:** clinical laboratory overuse, clinical necessity, clinical chemistry, test ordering

## Abstract

Diagnostic laboratory tests are frequently overused in healthcare entities, leading to an increased strain on laboratory resources, additional workload, and wastage of resources. Continuous monitoring of test ordering behavior is crucial to evaluate clinical necessity. This cross-sectional study aimed to estimate the necessity of ordering clinical chemistry tests in the cardiology clinic of a tertiary center in Saudi Arabia. We retrieved medical records of patients diagnosed with cardiovascular problems admitted at the cardiology clinic in 2020. The frequency and percentages of the ordered tests were calculated upon admission and follow-up, and the difference between necessary and unnecessary tests was compared for each category. Test ordering assessment included cardiac, renal, and liver functions, blood gases, thyroid and diabetic profile, iron indices, hormones, water and electrolytes, and inflammatory markers. The results showed a large number of clinical chemistry tests ordered without clinical necessity. While the number of necessary tests was significantly higher than that of unnecessary tests, 21% of the tests ordered between June-December 2021 at the center were unnecessary. Further studies are necessary to identify driving factors and develop strategies to reduce the overutilization of diagnostic laboratory tests in clinical practice. Eliminating this phenomenon will reduce the risk of unnecessary medical interventions and associated costs, improve patient outcomes, and reduce the overall burden on the healthcare system.

## INTRODUCTION

The overuse of unnecessary diagnostic tests is a widespread practice in hospitals, leading to a waste of valuable medical resources and potentially exposing patients to unnecessary risks. According to Frank H. Wians, unnecessary diagnostic tests are defined as "any test for which the results are not likely to be 'medically necessary' in the appropriate management of the patient's medical condition" [1]. In recent years, there has been a substantial increase in the number of vitamin tests ordered, particularly in developed countries. For example, in the Netherlands, vitamin B12 tests increased nearly sixfold between 2004 and 2014, and in the USA, vitamin D was the fifth most common laboratory test in 2016, costing 350$ million [2]. Studies suggest that as much as 60-70% of diagnostic tests may be doubtful of clinical importance [3], and over-ordering these tests can lead to patient distress and chances of false-positive test results [4].

Physician test-ordering behavior is a significant contributor to this phenomenon, which may be influenced by various factors, such as uncertainty, lack of experience, inadequate educational feedback, and unawareness of test costing [5]. A cross-sectional survey of hospital clinicians found that the strongest reason for ordering unnecessary tests was due to habitual behavior [6]. Beliefs that other physicians want the tests performed, medico-legal worries, concerns about surgical delays or cancellations, adherence to practice tradition, and a lack of awareness regarding evidence and guidelines were identified as contributing factors to ordering unnecessary preoperative tests [4]. A study at Nassau University Medical Center revealed that 50 patients received orders for prothrombin and partial thromboplastin coagulation tests without clear indication, causing unnecessary costs of $2434 [7]. In the Trent region, UK, a two-year study found that routine investigations could be waived after initial diagnosis, leading to an annual saving of £1.25 million [8]. Another study reviewing published literature from January 2012 to May 2019 estimated that a quarter of healthcare spending in the US is wasted [9]. Another study examining the John Hopkins overuse index of 375 areas in the US reported a regional systemic overuse of healthcare resources [10]. The availability of diagnostic test resources in Europe could be a driving factor that increases the inappropriate overuse of diagnostic laboratories [11]. Likewise, other scientists reported unnecessary testing, indicating that auditing and clinical discussions could help reduce unnecessary testing [12]. Moreover, another study, which analyzed data on beneficiary's insurance claims in the US between 2011 and 2015, reported a systemic overuse of medical care resources, especially in urban areas [13].

This study aimed to assess the extent of over-ordering unnecessary clinical chemistry laboratory tests and identify contributing factors related to previous research. Additionally, the study aimed to contribute to the development of future protocols and guidelines to reduce unnecessary testing and optimize resource utilization in the clinical setting.

## Material and Methods

This cross-sectional research was conducted to assess the potential overuse of clinical chemistry laboratory tests ordered for patients with cardiovascular problems at a tertiary center in the Eastern province of Saudi Arabia in 2020. The study was reviewed and approved by the institutional review board (IRB) at Imam Abdurrahman bin Faisal University (IRB-2021-3-181). All patients’ data were filtered so that personal identifying information was masked to protect patients’ privacy and confidentiality.

After obtaining ethical approval, we retrieved the medical records of patients admitted to the cardiology unit at the targeted center in 2020. We analyzed all related data from June to December 2021. A clinician with 20 years of experience in cardiovascular medicine and a medical doctorate reviewed all tests ordered for the study sample on admission and during follow-up visits to evaluate their clinical necessity. Only patients diagnosed with cardiovascular problems and referred to the clinical chemistry laboratory were included in the sample. Patients who had been referred to other clinics were excluded from the analysis.

The patient’s age and gender were recorded, and laboratory tests were categorized according to the data collection sheet. The laboratory tests included the main clinical chemistry panels, such as renal, liver, cardiac, diabetic, and lipid profiles, and miscellaneous tests to encompass special clinical chemistry tests. The data files were accessed via the information system of the cardiology unit. Diagnostic tests were either mentioned by name or category according to the data extracted from the system. Data were then filtered and reviewed by the appointed cardiologist to assess the clinical necessity of test ordering on admission and follow-up visits based on the clinical history and remarks registered into the system.

### Statistical analysis

The master data file was coded to convert categorical variables to continuous ones to perform comparative statistics. Demographic variables were summarized, and the percentages and frequencies of males and females were calculated. The mean age of both groups was then compared using Student’s t-test. Also, percentages of normal and abnormal laboratory tests on admission and follow-up of patients were calculated. The comparison between the number of necessary and unnecessary tests (ordered upon follow-up) was done using the binominal test. Differences were considered statistically significant at p ≤ 0.05. Statistical analysis was performed using the SPSS package (version 22).

## Results

The study sample included 387 males and 274 females, with mean ages of 58.15+14.1 and 57.27+16.2, respectively. There was no statistically significant difference between the mean ages of both groups. The age range of the study sample was 14-98 years (Table 1).

**Table 1:** Demographic characteristics of the study population

Gender	Frequency	Percent
**Male**	387	58.5
**Female**	274	41.5
**Age**	**(mean+SD)**	**P-value**
**Male**	58.15+14.1	0.458
Female	57.27+16.2	

### Frequency of laboratory tests ordered

**Figure 1:**
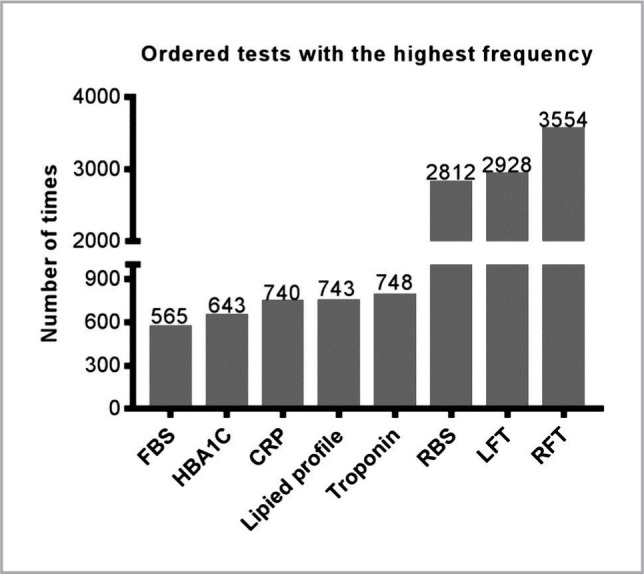
Frequency of clinical chemistry tests on patients' follow-up.

Table 2 shows the frequency of laboratory tests ordered on the admission of patients and on follow-up visits. The prevalence of each test was presented as a number and percentage, which was calculated for both normal and abnormal tests. Results showed that renal function tests (RFT) and liver function tests (LFT) were the most frequently ordered tests on follow-up visits, followed by random blood sugar (RBS), troponin, lipid profile tests, c-reactive protein (CRP), HBA1c, and fasting blood sugar (FBS), as summarized in Figure 1. Likewise, the number of RFT and LFT were also high on admission, but the number was lower compared to follow-up visits.

**Table 2:** Frequencies of the ordered diagnostic tests upon admission and follow-up.

	Lab Test	Results	Status	
			At admission	Follow-up
**Cardiac function tests**	Troponin	Normal	28 (43.8%)	378 (51%)
		Abnormal	36 (56.3%)	370 (49%)
	CPK	Normal	10 (30.3%)	72 (25%)
		Abnormal	23 (69.7%)	218 (75%)
	RFT	Normal	57 (14.8%)	495 (14%)
		Abnormal	327 (85.2%)	3059 (86%)
	LFT	Normal	40 (10.9%)	316 (11%)
		Abnormal	326 (89.1%)	2612 (89%)
	Lipid profile	Normal	19 (14.8%)	179 (24%)
		Abnormal	109 (85.1%)	564 (76%)
**Blood gas analysis**	Arterial Blood Gas	Normal	1 (11.1%)	14 (5%)
		Abnormal	8 (88.9%)	269 (95%)
	Venous Blood Gas	Normal	0 (0%)	23 (7%)
		Abnormal	18 (100%)	312 (93%)
**Thyroid profile**	Free T3	Normal	12 (85.7%)	73 (81%)
		Abnormal	2 (14.3%)	17 (19%)
	TSH	Normal	24 (92.3%)	144 (87%)
		Abnormal	2 (7.7%)	22 (13%)
**Diabetic profile**	RBS	Normal	34 (27.9%)	1152 (41%)
		Abnormal	88 (72.1%)	1660 (59%)
	FBS	Normal	17 (17.3%)	71 (13%)
		Abnormal	81 (82.7%)	494 (87%)
	Hb-A1c	Normal	12 (8.7%)	94 (15%)
		Abnormal	126 (91.3%)	549 (85%)
**Iron Indices**	Iron	Normal	4 (25%)	24 (26%)
		Abnormal	12 (75%)	69 (74%)
	TIBC	Normal	4 (21.1%)	211 (6%)
		Abnormal	15 (78.9%)	3343 (94%)
	Ferritin	Normal	10 (83.3%)	66 (50%)
		Abnormal	2 (16.7%)	66 (50%)
**Inflammatory markers**	CRP	Normal	5 (7.5%)	42 (6%)
		Abnormal	62 (92.5%)	698 (94%)
	Pro-calcitonin	Normal	5 (62.5%)	68 (41%)
		Abnormal	3 (37.5%)	99 (59%)
**Hormones**	Cortisol	Normal	1 (100%)	10 (43%)
		Abnormal	0 (0%)	13 (57%)
**Water & electrolytes**	Osmolality	Normal	1 (6.3%)	31 (32%)
		Abnormal	15 (93.8%)	65 (68%)

### Comparison between necessary and unnecessary-ordered tests

**Figure 2:**
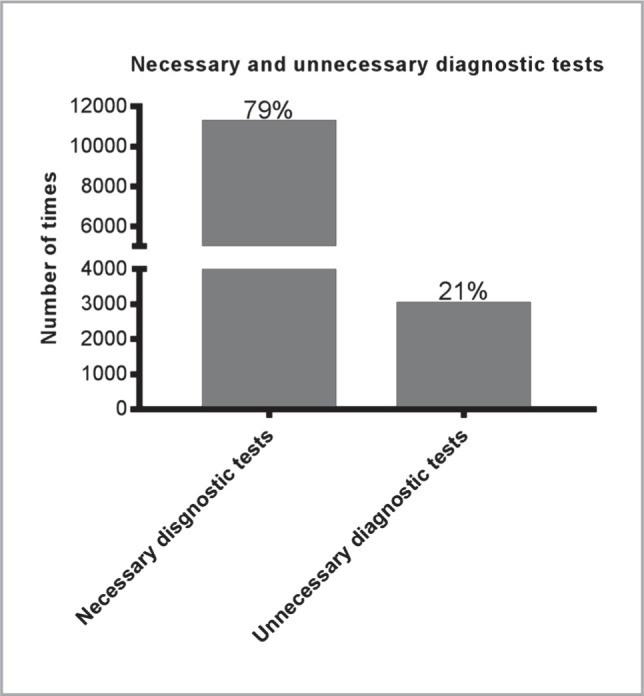
Diagnostic tests ordered with and without clinical necessity.

Table 3 presents the number of times each test (category) was ordered necessarily or unnecessarily and the statistical difference between them. Although the number of necessary tests ordered was statistically higher than the unnecessary tests (p ≤ 0.05 for most comparisons), many tests were ordered without clinical need. These include cortisol, which was ordered 50% of the time unnecessarily. Also, troponin and creatine phosphokinase (CPK) were ordered 43% of the time unnecessarily. Osmolality was requested unnecessarily 29% of the time, pro-calcitonin 28% of the time, random blood sugar (RBS) 26% of the time, and RFT and LFT 23% and 20% of the time, respectively. Overall, 21% of laboratory tests were unnecessarily ordered in the cardiology clinic, totaling 3005 tests. This indicates that almost one-fifth of the ordered tests were not clinically necessary. Figure 2 illustrates the numbers and percentages of necessary and unnecessary ordered diagnostic tests.

**Table 3:** Comparison between necessary and unnecessary ordered laboratory tests.

	Lab Test	Unnecessary n (%)	Necessary n (%)	P-value
**Cardiac function test**	Troponin	317 (43)	415 (57)	≤0.05*
	CPK	118 (43)	158 (57)	≤0.05*
	RFT	826 (23)	2692 (77)	≤0.05*
	LFT	577 (20)	2305 (80)	≤0.05*
	Lipid profile	42 (6)	681 (94)	≤0.05*
	Arterial Blood Gas	49 (17)	232 (83)	≤0.05*
	Venous Blood Gas	43 (13)	279 (87)	≤0.05*
**Thyroid profile**	Free T3	6 (7)	82 (93)	≤0.05*
	TSH	12 (7)	150 (93)	≤0.05*
**Diabetic profile**	RBS	691 (26)	1960 (74)	≤0.05*
	FBS	10 (2)	539 (98)	≤0.05*
	HbA1c	40 (6)	588 (94)	≤0.05*
**Iron Indices**	Iron	4 (4)	86 (96)	≤0.05*
	TIBC	28 (13)	182 (87)	≤0.05*
	Ferritin	19 (15)	108 (85)	≤0.05*
**Inflammatory markers**	CRP	144 (20)	577 (80)	≤0.05*
	Pro-calcitonin	42 (28)	109 (72)	≤0.05*
**Hormones**	Cortisol	11 (50)	11 (50)	1.00
**Water & electrolytes**	Osmolality	26 (29)	64 (71)	≤0.05*
	**Total**	**3005 (21)**	**11218 (79)**	≤0.05*

* – statistically significant difference at p≤0.05.

## Discussion

Continuous monitoring of healthcare facilities is crucial to ensure the efficient use of resources and prevent waste that can burden patients and staff. One area where resources can be overused is the clinical chemistry laboratory, where numerous diagnostic tests are performed to diagnose and monitor cardiovascular diseases. This cross-sectional study was conducted in a single healthcare center to identify potential deficiencies and formulate recommendations for the appropriate use of the clinical chemistry laboratory, which performs numerous diagnostic tests for diagnosing and monitoring cardiovascular diseases. The aim was to oversee unnecessary workload and improper management of resources.

Our results showed that many clinical chemistry diagnostic tests were ordered without a clinical necessity, accounting for 21% of all reviewed tests. This relatively high percentage observed in a single clinic highlights the significance of monitoring and optimizing the utilization of resources. A recent study identified several factors that contribute to the overuse of laboratory tests, including the interaction between primary care physicians and patients, physicians' experiences, fear of lawsuits, adherence to expert recommendations, and responsiveness to patients' requests based on their prior experience with laboratory test requests [14].

The overuse of diagnostic tests is a widely recognized issue in healthcare, as demonstrated by various studies from different countries. For example, a study conducted in an Australian hospital found that 60-70% of ordered clinical chemistry tests (potassium, lactate dehydrogenase (LD), and aspartate aminotransferase (AST)) and coagulation indices were not needed with absolute certainty, indicating possible overutilization of laboratory resources [3]. Another large cross-sectional study in the US reported the overuse of diagnostic tests in hospitals and confirmed the status of clinical laboratory overuse [15]. In a similar context, the diagnostic capacity, including imaging and laboratory testing, was evaluated in two Latin American states in centers that provide surgical care to ensure adequate and proper utilization of resources [16].

A recent systematic review has scrutinized research articles on clinical laboratory tests overuse [17]. The authors concluded that preoperative imaging and diagnostic tests due to non-specific back pain are the most frequently ordered low-value tests. They also noted a significant overuse of diagnostic laboratory tests in different healthcare settings [17]. Another systematic review focused on analyzing the factors contributing to over-testing and, consequently, overutilization of the clinical laboratory [18]. Fear of malpractice, clinician experience, sense of obligation to patients’ requests, and financial interest in test ownership are all factors reported as directly influencing the over-ordering of tests by clinicians. Other factors described as “extrinsic” include pressure from patients, ease of access to the test during day shifts, pressure from colleagues, pre-emptive testing, and advances in diagnostic techniques [18].

The literature on this topic confirmed the importance of continuous and meticulous monitoring of diagnostic tests overutilization to oversee unnecessary expenditures, undesired workloads, and waste of resources. Also, assessing test ordering capacity could help identify factors contributing to this issue and assess the need for clinical training when/wherever needed.

## Conclusion

The findings of this study highlight the potential for overutilization of diagnostic tests in a single healthcare center, with 21% of all ordered clinical chemistry tests found to be unnecessary. This reinforces the importance of regularly evaluating test ordering practices in healthcare settings to identify contributing factors to overuse and establish guidelines and policies to minimize this issue. Other studies have reported similar findings and highlighted the need for addressing factors such as fear of malpractice, clinician experience, and pressure from patients, among others, to reduce unnecessary testing. By addressing these factors, healthcare centers can help minimize the burden on patients and staff and save valuable resources. This study provides important data for future protocols and guidelines to improve the appropriate utilization of diagnostic tests in clinical settings.

## Data Availability

The data used to support the findings of this study are included in the article.
